# Aerosol Delivery of Lung Surfactant and Nasal CPAP in the Treatment of Neonatal Respiratory Distress Syndrome

**DOI:** 10.3389/fped.2022.923010

**Published:** 2022-06-15

**Authors:** Frans J. Walther, Alan J. Waring

**Affiliations:** ^1^Department of Pediatrics, David Geffen School of Medicine, University of California, Los Angeles, Los Angeles, CA, United States; ^2^Lundquist Institute for Biomedical Innovation at Harbor-UCLA Medical Center, Torrance, CA, United States; ^3^Department of Medicine, David Geffen School of Medicine, University of California, Los Angeles, Los Angeles, CA, United States

**Keywords:** surfactant, nasal CPAP, non-invasive ventilation, aerosol delivery, nebulization, neonatal respiratory distress syndrome, vibrating mesh nebulizers

## Abstract

After shifting away from invasive mechanical ventilation and intratracheal instillation of surfactant toward non-invasive ventilation with nasal CPAP and less invasive surfactant administration in order to prevent bronchopulmonary dysplasia in preterm infants with respiratory distress syndrome, fully non-invasive surfactant nebulization is the next Holy Grail in neonatology. Here we review the characteristics of animal-derived (clinical) and new advanced synthetic lung surfactants and improvements in nebulization technology required to secure optimal lung deposition and effectivity of non-invasive lung surfactant administration. Studies in surfactant-deficient animals and preterm infants have demonstrated the safety and potential of non-invasive surfactant administration, but also provide new directions for the development of synthetic lung surfactant destined for aerosol delivery, implementation of breath-actuated nebulization and optimization of nasal CPAP, nebulizer circuit and nasal interface. Surfactant nebulization may offer a truly non-invasive option for surfactant delivery to preterm infants in the near future.

## Introduction

Early respiratory failure in very preterm infants (respiratory distress syndrome, RDS) is caused by lung immaturity and not yet started production of lung surfactant (1). Mammalian lung surfactant, a mixture of phospholipids and four surfactant proteins, is produced by alveolar type 2 cells and consists of the essential phospholipids dipalmitoyl phosphatidylcholine (DPPC) and phosphatidylglycerol and the surfactant proteins SP-A, SP-B, SP-C, and SP-D. DPPC contributes to the formation of a rigid interfacial film that reduces surface tension in the alveoli to low values during dynamic compression, whereas fluid phospholipids and neutral lipids significantly improve film spreading. SP-A and SP-D play important roles in native immunity, whereas SP-B and SP-C enhance the absorption of phospholipids into the air-water interface and optimize surface tension reduction. SP-B is a crucial protein component of lung surfactant and its deficiency due to genetic causes is lethal in both animals and humans (2). SP-C deficiency can cause childhood interstitial lung disease with variable disease severity in infancy (3).

After studies in preterm animals in the 1980s demonstrated a rapid improvement of oxygenation and lung compliance after intratracheal bolus instillation of an animal-derived lung surfactant, its general clinical use, in combination with technological advances in infant ventilators and perinatal care, has dramatically improved survival of very preterm infants (4). Limited availability, high production costs, and scarcity of animal sources have led to the design and development of synthetic lung surfactant formulations, consisting of SP-B and/or SP-C peptide mimics in a phospholipid mixture (5). Similarly, respiratory care of very preterm infants with RDS has been shifting away from intubation and mechanical ventilation to nasal continuous positive airway pressure (nCPAP) (6, 7) to avoid invasive mechanical ventilation lung injury and induction of bronchopulmonary dysplasia, a major pulmonary complication among extreme preterm infants that is often accompanied by neurodevelopmental disabilities. Although surfactant can be instilled intratracheally during spontaneous breathing on nCPAP by inserting a thin catheter in the trachea and thus does not require intubation, this so-called less invasive surfactant administration (LISA) requires advanced technical skills to be able to pass a catheter through the vocal cords (8). Aerosol delivery of surfactant would therefore complement non-invasive ventilatory management of preterm infants with RDS. Technical advances in non-invasive aerosol delivery of surfactant are an interplay of nebulizer technology, appropriate dry powder or liquid animal-derived or synthetic lung surfactant, an optimum delivery route, and lung dose.

Whereas surfactant replacement therapy has become standard of care for surfactant deficiency in preterm infants, it can also enhance the treatment of surfactant dysfunction disorders in infants and children with acute respiratory distress syndrome (ARDS). Meconium aspiration syndrome is an example of surfactant dysfunction in (near) term infants (9), but ARDS can also occur in infants and children who have experienced trauma, pneumonia, aspiration, or immune compromise (10). Here, we review current developments in the surfactant field that are important for treatment of RDS in preterm infants.

## Surfactant

Currently available surfactant can be divided into clinical and synthetic lung surfactant preparations. Clinical surfactants are derived from bovine (Survanta^®^, Infasurf^®^, Alveofact^®^) or porcine (Curosurf^®^) lungs and consist of phospholipids and small quantities (~1 weight%) of the hydrophobic proteins SP-B and SP-C. SP-A and SP-D are hydrophilic proteins and are lost during extraction of the lavage material or minced animal lung tissue. Clinical surfactants were preceded by the first generation of synthetic lung surfactants, i.e., ALEC^®^ (11), a mixture of DPPC and egg phosphatidylglycerol (PG), and Exosurf^®^ (12), a mixture of DPPC with hexadecanol and tyloxapol. These pure phospholipid-based preparations lacking SP-B and SP-C were quickly overshadowed by the introduction of clinical surfactants with a rapid and more consistent improvement in lung compliance after intratracheal administration. The second generation of synthetic lung surfactants contained SP-B and/or SP-C peptide mimics, such as the SP-B analog KL4 [Surfaxin^®^ (13) and Aerosurf^®^ (14)] and recombinant hSP-C [Venticute^®^ (15)], but their efficacy was no match with the animal-derived surfactants and demonstrated the complex 3-D structure of native SP-B and the propensity of SP-C for amyloid formation of its transmembrane region. Advances in the design, development, and production of SP-B and SP-C peptide mimics and phospholipid mixtures subsequently led to the creation of new surfactant formulations with surface activity equivalent to the clinical surfactants.

*Human SP-B* is a hydrophobic homodimer of two 79 amino-acid polypeptide chains that are linked by disulfide bonds. Each monomer has a molecular weight of 8.7 kDa and consists of 5 α-helices stabilized by three intramolecular disulfide bonds and the two monomers are connected with one interchain disulfide. The N- and C-terminal peptides of SP-B enhance adsorption and surface spreading of phospholipids in a similar way as native SP-B (16) and these characteristics were used to design and develop the SP-B peptide mimics Mini-B, Super Mini-B and B-YL (5). Mini-B is a 34 amino acid peptide that incorporates the N-terminal (residues 8–25) and C-terminal (residues 63–78) α-helices of native SP-B, joined with a customized turn and cross-linked with two adjoining disulfide bonds to form an α-helix hairpin (17). Super Mini-B and B-YL contain the hydrophobic N-terminal insertion sequence of SP-B (residues 1–7) and are 41 amino-acid peptides (18, 19). B-YL has no disulfide bonds after replacing the cysteine residues of Super Mini-B with tyrosine and is less sensitive to oxidation by replacing the methionine residues with leucine. These SP-B peptide mimics are highly stable in phospholipid mixtures and exert excellent surface activity *in vitro* and in animal models of surfactant deficiency (20, 21).

*Human SP-C* is an extremely hydrophobic 35 amino-acid protein with a molecular weight of 4.2 kDa. It consists of an α-helix (residues 9–34) and two palmitoyl chains bound to cysteines in positions 4 and 5 *via* labile thioester bonds. Several large multicenter studies using recombinant SP-C (rhSP-C) without palmitoyl chains (Venticute) demonstrated that palmitoylation of SP-C is essential for optimal surface activity. Johansson et al. (22) solved this problem by replacing the palmitoylated cysteines with serine and preserving the transmembrane helical domain by substituting leucines for valines (rSP-C33Leu). SP-C33Leu has shown to have good *in vitro* and *in vivo* surface activity, especially in combination with a SP-B peptide mimic (23). Stabilization of the α-helix of SP-C with intrapeptide salt-bridges (“ion locks”) (24) and use of helical poly-N-substituted glycines (peptoids) (25) have also created stable SP-C mimics with elevated *in vitro* and *in vivo* surface activity.

Availability of these highly functional SP-B and SP-C mimics has led to the development of third generation synthetic lung surfactants when mixed in surfactant phospholipids. CHF5633 (Chiesi Farmaceutici, Parma, Italy) is a liquid combination of 0.2% Mini-B and 1.5% SP-C33 in a 1:1 DPPC:POPG mixture (23) and has recently shown clinical efficacy and safety in preterm infants with neonatal RDS (26–28) if administered intratracheally. Minisurf^®^ is a liquid synthetic surfactant based on 3% Super Mini-B in a 5:3:2 DPPC:POPC:POPG (mole:mole) mixture (Molecular Express Inc., Rancho Dominguez, CA 90220) and is in a preclinical stage. Both CHF5633 and Minisurf have demonstrated *in vitro* and *in vivo* surface activity similar to animal-derived clinical surfactants. Aerosurf (Surfaxin for inhalation) is a combination of KL4 surfactant and aerosolization technologies from Windtree Therapeutics (Warrington, PA 18976). The lyophilized KL4 surfactant needs to be reconstituted with sterile water prior to non-invasive aerosol delivery with the Aerosurf Delivery System (ADS), which uses novel heated capillary-based aerosol generator (CAG) technology (29). Aerosurf is currently being tested in clinical studies.

The challenge of pulmonary drug delivery is to achieve a high lung deposition of inhaled aerosols. The fate of inhaled particles is determined by their physicochemical properties, deposition site in the airways and biological defenses, such as mucociliary transport and the presence of resident airway macrophages. Solid state drugs have improved chemical stability and this is true for lung surfactant too. Whereas most clinical and synthetic lung surfactant formulations are liquids and consist of SP-B and/or SP-C (peptide mimics) in a phospholipid mixture, dry powder surfactant undergoes spray-drying steps aimed at controlling particle size, density, shape, charge, solid state, and hygroscopicity in order to manipulate agglomeration and powder blending (30). Formulation strategies may include the addition of hygroscopic excipients, such as sugars (e.g., lactose, trehalose, and mannitol) and sodium chloride, and/or dispersion enhancers, such as l-leucine, to alter particle-particle interaction forces and improve the deagglomeration of the fine particles (31, 32). These newer micron-sized dry powder synthetic lung surfactant formulations, such as B-YL:Trehalose surfactant (21, 31), are highly surface active, possess the required aerosolization characteristics, and are biophysically stable. In combination with an appropriate nebulizer, both liquid and dry powder clinical and synthetic surfactant formulations have great potential to resolve surfactant deficiency *in vivo*.

## Nebulization

Aerosol delivery of surfactant has a 30-year history (33), but early studies in preterm infants with clinical surfactant in the 1990s with Alveofact (34), Exosurf (35), and Curosurf (36) did not meet expectations. The gradual shift to non-invasive ventilation of very preterm infants (37) and LISA via a thin intratracheal catheter (8) sparked enthusiasm for the pursuit of fully non-invasive surfactant treatment on two fronts, nebulization devices and appropriate (synthetic) lung surfactant formulations.

Very preterm infants are obligate nose breathers and have a low functional residual capacity, a high and variable respiratory rate, low tidal volumes and small airways (38). In healthy infants, nasal airway resistance accounts for nearly half of the respiratory resistance and turbulent flow in the upper airways is a major causative factor for drug loss through impaction (39). These characteristics pose a major challenge to aerosol delivery of surfactant because they are associated with a low lung deposition of inhaled drugs. However, lung deposition is also affected by the type of non-invasive ventilation (NIV) used, the choice of aerosol generator and its configuration within the ventilation circuit, the choice of surfactant composition and its aerosol particle size distribution, and, last but not least, the interface between the delivery system and the preterm infant. Attention to all these factors is necessary to optimize aerosol delivery of lung surfactant and its clinical efficacy (38).

### Non-invasive Ventilation

Current standards of respiratory care for very preterm infants with RDS aim at reducing bronchopulmonary dysplasia through early use of nCPAP and, if necessary, less invasive surfactant administration (40). Other modes of NIV, i.e., nasal intermittent positive pressure ventilation (NIPPV), biphasic positive airway pressure (BiPAP), and high-flow nasal cannula (HFNC) are less often used than nCPAP. CPAP failure in very preterm infants is associated with a higher risk of poor outcome and is an indication for intubation and invasive mechanical ventilation (41). Widespread use of nCPAP has led to the development of a multitude of shapes and sizes of face masks and nasal prongs for preterm and term infants. Differences in their geometrics can influence airflow characteristics, especially airway resistance, and ultimately affect the lung surfactant dosage necessary to elucidate a clinical effect (42). Air leakage continues to require outmost attention, but is not only limited to face masks and nasal prongs, but may also concern air leakage through the mouth.

### Nebulizers

Nebulizers are one of the oldest clinically used aerosol generating systems and form a mist of micronized fine particles of a solid or liquid active pharmaceutical ingredients (API) that can be inhaled into the lungs (43). Particle size is expressed as mass median aerodynamic diameter (MMAD) and lung surfactant particles should be within a 1 to 5 μm range (44).

Pressurized metered-dose inhalers (pMDIs) and jet, ultrasonic, and vibrating mesh nebulizers are the most commonly used aerosol delivery devices in infants (45). A pMDI produces in a short time period an aerosol cloud that moves at high speed and requires active inhalation. pMDIs are therefore more often used in children with respiratory conditions such as asthma and cystic fibrosis and who can coordinate actuation and inhalation. *In vitro* and *in vivo* studies have shown that vibrating-membrane nebulizers (VMNs) outperform jet and ultrasonic nebulizers in aerosol delivery of liquid surfactant formulations to ventilated newborn infants. Tiemersma et al. (45) compared two prototypes of a VMN, a jet nebulizer and a pressurized metered-dose inhaler (pMDI) with a holding chamber in an upper airway (nose-throat) model of a 32-week preterm infant (1,750 g). Lung dose was 18.0–20.6, 1.5, and 6.8%, respectively. This has led to a shift away from jet nebulizers to VMNs in animal and clinical studies (Tables 1, 2). Synchronization of aerosol delivery and spontaneous respiration can further enhance lung dose as recently shown in ventilated, surfactant-deficient juvenile rabbits and in a porcine ARDS model using liquid bovine surfactant and a novel breath-actuated small particle VMN with photo-defined aperture plate (PDAP) technology (Aerogen Pharma, San Mateo, CA) placed between the Y- connector of the ventilator circuit and the endotracheal tube (63, 64). Synchronization of surfactant delivery was managed by a flow sensor in the inspiratory line of the ventilator and minimized surfactant loss during expiration. Estimation of the lung dose *in vitro* suggested an efficiency of ~65%. Developments in this field are going fast and Wiegandt et al. (65) recently produced a nasal prong with an integrated miniaturized aerosol valve that allows breath-triggered drug release directly inside the nasal prong with a short response time.

**Table 1:** Summary of aerosolized surfactants and nebulizers utilized in studies in animals supported with nasal CPAP (nCPAP) ventilation.

**Author**	**Model**	**Nebulizer**	**Surfactant**	**Control**	**Measurements**	**Conclusion**
Rahmel et al. (46)	Preterm lambs	Continuous Powder Aerosolization system (63)	rSP-C surfactant with Sm_2_O_3_ label	Humidified air	Blood gases, hematology, histopathology, minute volume, lung deposition	Safe; not effective; highest lung deposition if surfactant flow was synchronized with spontaneous breathing
Hütten et al. (47)	Preterm lambs	eFlow-Neos VMN	Curosurf	Saline	Oxygenation, lung gas volumes and DSPL pool size and distribution	Three hours of surfactant in humidified air (861 mg/kg) improved oxygenation and lung function, but 60 min (437 mg/kg) and 0 min (229 mg/kg) did not
Linner et al. (48)	Newborn piglets	eFlow-Neos VMN	Curosurf 200 mg/kg	Nebulization and IT instillation	Lung deposition measured by gamma scintigraphy	Median lung deposition: 5% *via* mask, 14% *via* prongs, and 45% *via* endotracheal tube (*p* <0.05)
Milesi et al. (49)	Preterm lambs	Supraglottic atomizer, synchronized with inspiration	Curosurf 200 mg/kg	CPAP alone	Blood gases, electrical impedance tomography and carotid blood flow	Safe, improved oxygenation and ventilation homogeneity compared with CPAP alone; lung deposition of 32% (22–43%).
Bianco et al. (50)	Lavaged rabbits	eFlow-Neos VMN	Curosurf 100–600 mg/kg	nCPAP alone and Curosurf 200 mg/kg IT	Oxygenation and lung mechanics. Lung deposition (exogenous alveolar DPPC)	Improved oxygenation vs. nCPAP alone; 200 mg/kg and 400 mg/kg doses had equivalent improved oxygenation and lung mechanics as IT surfactant
Cunha-Goncalves et al. (51)	Newborn piglets	eFlow-Neos VMN	Curosurf 200 mg/kg with technetium label	Prone vs. supine vs. right side up vs. left side up	Lung deposition measured by gamma scintigraphy	Lung surfactant deposition was highest in the prone position (32.4 ± 7.7%)
Rey-Santano et al. (52)	Lavaged newborn piglets	eFlow-Neos VMN	Curosurf 400 mg/kg	CPAP alone and CPAP-INSURE	Gas exchange, lung mechanics, lung histology	Safe and effective in reducing the risk of respiratory failure in the 72 h after treatment similar to CPAP-INSURE
Rey-Santano et al. (53)	Lavaged newborn piglets	eFlow-Neos VMN	Curosurf 100, 200, 400, and 600 mg/kg	CPAP alone and CPAP-INSURE	Gas exchange, lung mechanics, lung injury, intrapulmonary shunting, carotid blood flow	Nebulization of 200 or 400 mg/kg of Curosurf improved pulmonary outcomes similar to CPAP-INSURE
Gregory et al. (29)	Non-human primates	Aerosurf Delivery System (ADS)	Aerosurf (KL4) with technetium label	None	Lung deposition measured by gamma scintigraphy	Lung deposition was 11%, regional deposition within the lung was generally homogeneous
Nord et al. (54)	Newborn piglets	eFlow-Neos VMN	Curosurf 600 mg/kg with technetium label	Curosurf 200 mg/kg IT	Lung deposition	Mean ± SD lung dose of phospholipids was 138 ± 96 mg/kg with nebulization, and 172 ± 24 mg/kg with instillation (*p* = 0.42)
Nord et al. (55)	Newborn piglets	eFlow-Neos VMN	Curosurf 200 mg/kg with technetium label	Curosurf 200 mg/kg with technetium label by NIPPV	Oxygenation and lung deposition	Mean ± SD lung deposition was 15.9 ± 11.9% with nCPAP and 21.6 ± 10% with NIPPV (*P* = 0.20) Blood gases were comparable in both groups.
Nord et al. (56)	Newborn piglets	Atomizing catheter in the upper pharynx, synchronized with inspiration	Curosurf 200 mg/kg with technetium label	Curosurf 200 mg/kg IT	Lung deposition	Median (range) lung deposition was 40% (24–68%) after atomization and 87% (55–95%) after instillation (*p* <0.001)
Walther et al. (31)	Preterm lambs	Capsule-based low flow aerosolization chamber (LFAC)	Dry powder synthetic lung surfactant 100 or 200 mg/kg	Curosurf 200 mg/kg IT	Particle sizing, oxygenation and lung function	Stabilization of spontaneous breathing and oxygenation and lung volumes comparable to IT Curosurf control

*DPPC, dipalmitoylphosphatidylcholine; IT, intratracheally; NIPPV, nasal intermittent positive pressure ventilation; SD, standard deviation; VMN, vibrating mesh nebulizer. The eFlow Neos VMN is a product of PARI GmbH, Starnberg, Germany*.

**Table 2:** Summary of aerosolized surfactants and nebulizers in clinical studies of preterm infants supported with nasal CPAP (nCPAP).

**Authors**	**# Infants**	**GA, weeks**	**Age, h**	**Surfactant**	**Dosage**	**Nebulizer**	**Nebulizer position**	**NIV**	**Controls**	**Outcome**
Jorch et al. (34)	20	28–35	1–7	Alveofact	2x 150 mg/kg	Jet	Y piece	Bubble pharyngeal CPAP	No	Improved oxygenation; 6/20 (30%) required intubation
Arroe et al. (35)	22	23–36	<72	Exosurf	108,216 or 432 mg	Sidestream 45 Jet	Inspiratory limb	nCPAP	No	No adverse effects; no improved oxygenation; 8/22 (36%) required intubation
Berggren et al. (36)	32	27–34	<36	Curosurf	480 mg/kg	Aiolos Jet	Inspiratory limb	nCPAP	nCPAP alone	Safe, but no beneficial effects of aerosolized surfactant
Finer et al. (14)	17	28–32	<1	Aerosurf	72 mg, 3 retreatments allowed <48 h	Aeroneb Pro VMN	Y piece	nCPAP	No	Well tolerated; transient desaturations during dosing; 29% required intubation
Minocchieri et al. (57)	64	29^0/7^-33^6/7^	<4	Curosurf	100 mg/kg, repeat after 12 h	eFlow–Neos VMN	Y piece	Bubble nCPAP with FiO_2_ 0.22–0.3	Bubble nCPAP alone	11/32 (34%) nebulized infants vs. 22/32 (69%) CPAP controls were intubated <72 h, significance limited to 32^0/7^-33^6/7^ weeks GA, no major adverse effects
Sood et al. (58)	17	24^0/7^-36^6/7^	<72	Survanta	100 or 200 mg/kg and 2 dilutions at (12.5 or 8.3 mg/ml)	MiniHeart Lo–Flo Jet	Inspiratory limb	11 infants on NIPPV, 4 on CPAP, and 2 on HFNC	100 vs. 200 mg	No adverse effects; 5/17 (29%) infants were intubated <72 h; 13 (76%) got 2 doses; no change in FiO_2_
Cummings et al. (59)	457	23–41	<12	Infasurf	210 mg/kg up to 3x <72 h	Solarys Jet with modified pacifier (0.20 ml/min)	Inside the mouth	NIV with FiO_2_ 0.25–0.40	Usual care	Intubation <96 h: 26% in the aerosol group vs. 50% in the controls (*P* <0.0001); respiratory outcomes up to 28 days of age were no different
Sood et al. (60)	149	24^0/7^-36^6/7^	<24	Survanta	100 or 200 mg at (12.5 or 8.3 mg/ml), 1 redosing allowed	MiniHeart Lo–Flo Jet and AeroNeb Solo VMN	Inspiratory limb	NIV with FiO_2_ ≥25%, PEEP ≥4 cmH_2_O or flow rate ≥2 LPM	No	15 infants (10%) required intubation <72 h; adverse events included surfactant reflux (18%) and desaturations (11%)
Nasl intermittent Dani et al. (61)	129	28^0/7^-32^6/7^	<12	Curosurf	200 vs. 400 mg/kg, 1 redosing allowed at 200 mg/kg	eFlow–Neos VMN	Y piece	nCPAP	nCPAP alone	No significant differences in the intubation rate <72 h and secondary respiratory outcomes; nasal prongs were not sealed; no safety concerns
Jardine et al. (62)	31	26^0/7^-30^6/7^	<2	Alveofact	216 mg/kg 1x in part 1 (*n* = 10) and up to 4x in part 2 (*n* = 21)	AeroFact VMN, synchronized with inspiration	Y piece	nCPAP	93 historic controls	Treatment failure in part 2 was 29% vs. 48% in controls (*p* = 0.10); no safety issues were identified

*FiO_2_, fraction of inspired oxygen; HFNC, high flow nasal cannula; LPM, liters per minute; NIPPV, nasal intermittent positive pressure ventilation; NIV, non-invasive ventilation; PEEP, positive end-expiratory pressure; VMN, vibrating mesh nebulizer*.

Aerosol delivery of dry powder surfactant can be achieved by generating an aerosol using the inspiratory air flow from the ventilator or by separately producing an aerosol and inserting it into the inspiratory air flow (66). Lung dose from dry powder inhalers is affected by humidity when ventilatory circuits are driven with warm and humified gas and this is why dry powder surfactant is formulated with hygroscopic excipients and/or a dispersion enhancer. We recently reported the feasibility of nasal delivery of dry powder synthetic lung surfactant with B-YL peptide as SP-B mimic and trehalose as excipient to preterm lambs with RDS who were supported with bubble nCPAP. A cylindrical low-flow aerosol chamber (LFAC) device (31) was placed below the Y of the nCPAP circuit and accommodated a perforated capsule with surfactant powder that was delivered at low flows (4–10 L/min) (31). The dry powder surfactant particles tended to aggregate upon delivery due to humidification of the oxygen respiratory gases in the circuit. Half of the lambs required a second dose of ~100 mg/kg of surfactant to reach oxygenation and lung gas volumes similar to the positive Curosurf controls.

Beyond nebulizer technology, location of the nebulizer in the ventilatory circuit and the characteristics of the CPAP interface (prongs or facemask) are important for its efficacy. Placing the nebulizer between the Y-piece and the CPAP interface achieves far higher lung doses than placement in the inspiratory limb way before the Y-piece. Depositional loss of nasally inhaled aerosols in infants has been investigated *in vitro* in a preterm nose-throat replica (67). Prong type (internal or external), configuration (single and dual), and insertion depth all affect lung delivery efficiency. Dual prongs at an insertion depth of 2 mm are probably best with upper airway losses of 15–20% (44). Various studies describe ingenious modifications of the nasal interface to improve aerosol delivery during nCPAP support, but these have not been tested *in vivo* yet (68).

## Findings from Animal and Clinical Studies

### Animal Studies

Table 1 shows an overview of animal studies in which non-invasive ventilatory support was provided with nCPAP and various clinical and synthetic surfactants were delivered by aerosolization (46–56, 69). Nebulization of surfactant was safe and major adverse effects were limited to reflux of surfactant and temporary desaturation during the procedure. Effectivity, measured by improvement in oxygenation and lung compliance, varied considerably and correlated with lung deposition which varied from 11 to about 40%. Doubling of the clinically used dosage of Curosurf (400 instead of 200 mg/kg) was well tolerated and possibly more effective, but tripling of the dosage (600 mg/kg) did not provide better results (50, 53, 54). Most animal studies opted for the use of VMN nebulizers, demonstrating the move away from jet nebulizers in newborns. Synchronizing surfactant delivery with inspiration led to higher lung deposition (49, 56) and therefore deserves further exploration. Older synthetic surfactants (29, 46) were less effective than clinical or newer synthetic lung surfactant (31).

### Clinical Studies

Early clinical studies with jet ventilators published in the 1990s indicated that surfactant nebulization was safe, except for surfactant reflux and desaturations, but clinical results did not meet expectations [Table 2, (34–36)]. The pilot study by Finer et al. (14) with a prototype VMN nebulizer has rekindled clinical studies on non-invasive surfactant administration in preterm infants supported with nCPAP (Table 2). Up so far, six published clinical studies have investigated whether non-invasive aerosol delivery of animal-derived (clinical) surfactant can prevent the need for early intubation for invasive mechanical ventilation and/or intratracheal surfactant instillation (57–62). Three studies showed a significant reduction in nCPAP failure (57, 59, 62), two studies (58, 60) did not recruit controls but their results pointed in the same direction, and one study (61) did not show any effectivity, possibly because of air leakage around the nasal prongs. The most recent published study (62) used a VMN nebulizer with breath-actuation of surfactant delivery and suggests that a double dose of clinical surfactant given at the right time of the respiratory cycle may be the way forward in non-invasive surfactant administration in preterm infants with RDS who are supported with nCPAP ventilation. Although this study is relatively small, its positive results warrant larger studies exploring this technology.

Both animal and clinical studies show the important role of currently available clinical surfactant preparations in aerosol delivery of surfactant therapy in infants. Newer dry powder and liquid synthetic surfactant formulations are catching up with clinical surfactants and may provide a more cost-effective therapeutic option than animal-derived (clinical) surfactants in the near future.

## Conclusion

Non-invasive surfactant delivery is currently the Holy Grail in neonatology, but its search is challenged by technical problems to optimize lung deposition of nebulized surfactant. The currently available evidence suggests that non-invasive nebulization of clinical and advanced synthetic lung surfactant with breath-actuated VMN technology and an appropriate nasal interface is a promising way forward and has great potential to become part of future non-invasive respiratory care for preterm and term infants. Ongoing research on nebulizer technology and its nasal interface should focus on optimization of the delivery route and lung dose of highly active lung surfactant synthetic formulations.

## Author Contributions

FW wrote and AW revised the manuscript. All authors contributed to the article and approved the submitted version.

## Funding

This work was supported by the Bill & Melinda Gates Foundation (INV-001227 and INV-040846). Under the grant conditions of the Foundation, a Creative Commons Attribution 4.0 Generic License has already been assigned to the author accepted manuscript version that might arise from this submission. The funders had no role in study design, data collection and analysis, decision to publish, or preparation of the manuscript.

## Conflict of Interest

The authors declare that the research was conducted in the absence of any commercial or financial relationships that could be construed as a potential conflict of interest.

## Publisher's Note

All claims expressed in this article are solely those of the authors and do not necessarily represent those of their affiliated organizations, or those of the publisher, the editors and the reviewers. Any product that may be evaluated in this article, or claim that may be made by its manufacturer, is not guaranteed or endorsed by the publisher.

## Abbreviations

ARDS: Acute respiratory distress syndrome
CPAP: Continuous positive airway pressure
DPPC: Dipalmitoylphosphatidylcholine
FiO_2_: Fraction of inspired oxygen
HFNC: High flow nasal cannula
INSURE: Intubation, surfactant extubation
IT: Intratracheally
LFAC: Low flow aerosolization chamber
LISA: Less invasive surfactant administration
LPM: Liters per minute
nCPAP: Nasal CPAP
NIPPV: Nasal intermittent positive pressure ventilation
NIV: Noninvasive ventilation
PEEP: Positive end-expiratory pressure
PG: Phosphatidylglycerol
pMDI: Pressurized metered-dose inhalers
POPC: 1-palmitoyl-2-oleoyl-sn-glycero-3-phosphocholine
POPG: 1-palmitoyl 2-oleyl phosphatidylglycerol
RDS: Respiratory distress syndrome
rhSP-C: Recombinant human SP-C
SD: Standard deviation
SP: Surfactant protein
VMN: Vibrating mesh nebulizer.
